# A Vital Question for Voluntary Hospitals

**Published:** 1917-11-10

**Authors:** 


### A VITAL QUESTION FOR VOLUNTARY HOSPITALS.

In our issue of August 25 we dealt with a speech of Colonel Bruce Vaughan, chairman of the board of management of King Edward VII.'s Hospital, Cardiff, who quoted Sir Garrod Thomas to the effect that many hospitals were largely staffed with unqualified men. In support of this Colonel Vaughan declared that "the resident staff in the Cardiff Hospital had not been so efficient as it should be. The present difficult and haphazard method of staffing the voluntary hospitals in the kingdom was a scandal, and ought to be inquired into and a remedy found without delay." This position was clearly serious and calculated to create grave and increasing dissatisfaction amongst the civilian population especially. It afforded startling evidence, too, of the very serious responsibility cast upon the managing bodies of the hospitals where this state of affairs prevailed, to a greater or less extent, and called for prompt action on their part, and indeed upon the part of hospital managers generally. There is no ground for panic, but there is ground for a thorough investigation into the facts, and unless the voluntary hospitals themselves take up this matter promptly and exhaustively, it may result in grievous injury, for which a statesmanlike policy and combined action, in conjunction with the Government and War Office, should be capable of providing a remedy before it is too late.

Allied to these questions are those which were raised at the recent meeting of the British Hospitals Association at the Westminster Hospital, though they relate not to the treatment of civilian patients but to the admission and treatment of sailors and soldiers, not as objects of charity in any sense, a point on which the whole nation has expressed a clear wish and view. That is to say, the nation is resolved that under no circumstances shall sailors and soldiers suffering from wounds or illness be admitted to a hospital or other medical institution except as the guests and at the cost of the State.

The Council of the British Hospitals Association has not so far met, in fulfilment of the chairman's pledge, to take action upon the resolutions adopted, with practical unanimity, at the meeting on October 19. It may be well, therefore, to point out the urgency of immediate action on Mr. Wade Deacon's part, which is increased, some consider, by the attitude members of the medical profession have taken up in regard to the questions dealt with by the meeting of hospital managers at Westminster Hospital. We hope no further delay may ensue, but that the Council concerned may be summoned at once to deal with the questions they have to consider, with a view to the resolutions resulting in practical and prompt action.

In our article of August 25, page 408, we pointed out that the lack of efficiency within the hospitals complained of by Colonel Bruce Vaughan and the representatives of working-class governors in connection with some of the large voluntary hospitals, could not constitute a danger or be injurious to the interests of the patients if the medical staff of every hospital were to co-operate, and so enable each institution to make adequate provision, whereby the maximum of efficiency in treatment could be guaranteed. We expressed the hope that without delay every hospital committee would look into this matter and call a conference with the members of the honorary medical staff within reach. The urgency of the questions involved, possibly owing to the holidays, does not seem to have been grasped by voluntary hospital committees and their medical staff, but the proof of it is to be found in the action taken on behalf of the Northumberland miners. A notice appears in the *Evening Standard* of the 6th instant that it is proposed, on behalf of the Northumberland miners, to ask Parliament to institute a Committee of Inquiry into the working of the hospital system of the county, on the ground that the care of civilians in sickness or accident can be no longer left to voluntary institutions, which even before the war were unable to meet the demands upon them. This action demonstrates the force and truth of what we have urged in these columns more than once in recent months, and will, we hope, ensure the adoption of the course we have ventured to suggest, so that every hospital may ascertain precisely how far the questions raised affect its working at the moment. Further, that steps may be taken to call a conference of the responsible representatives of the principal voluntary hospitals throughout the country, with a view to decide upon the course of action best calculated to meet the position adequately and promptly.

